# Gender Differences in Mental and Physical Health: Implications for Aging With a Traumatic Brain Injury

**DOI:** 10.1093/geroni/igab046.2666

**Published:** 2021-12-17

**Authors:** Emma Swinford

**Affiliations:** University of Missouri-Kansas City Institute for Human Development, Kansas City, Missouri, United States

## Abstract

Traumatic Brain Injury (TBI) is a major cause of disability and death in the U.S., and survivors often experience temporary or life-long health effects as a result of their injury. While risks and outcomes of fall-related TBI in older adults have been well-documented, the intersection of TBI-related health challenges and the experience of aging with a TBI is less well understood. This project explores gender differences in health outcomes among TBI survivors. A Needs Assessment survey was conducted in early 2020 with adult TBI survivors in Missouri (n=150). The mean age of respondents was 46 and 58% identified as male. Bivariate analyses reveal gender differences in health conditions among TBI survivors before and after injury. Significantly more males than females reported substance use disorder for alcohol (20.7% and 7.9%, p < .05) prior to injury, whereas twice as many females reported developing chronic pain after injury than males (68.3% and 31.0%, p < .001). Further, while about 21% of both male and female respondents reported experiencing other mental health conditions, such as anxiety, prior to injury, over 35% of males and almost 58% of females experienced mental health concerns after injury. Additionally, balance/mobility issues, sleep disorders, sensory issues, and cognitive challenges were frequently identified post-injury conditions. Co-morbidities impact our experiences, capabilities, and quality of life as we age. Policies and programs to support TBI survivors and their families may better address the co-occurring health conditions among TBI survivors by considering gender differences in the experience of aging with a TBI.

